# Plasma irisin in runners and nonrunners: no favorable metabolic associations in humans

**DOI:** 10.14814/phy2.12262

**Published:** 2015-01-19

**Authors:** Tamara Hew‐Butler, Kristin Landis‐Piwowar, Gregory Byrd, Max Seimer, Nicole Seigneurie, Brigid Byrd, Otto Muzik

**Affiliations:** Oakland University, Rochester, Michigan, USA; Wayne State University, Detroit, Michigan, USA

**Keywords:** Brown fat, energy expenditure, running

## Abstract

Irisin is a hormone which mimics the favorable metabolic effects associated with regular exercise, by converting subcutaneous white fat into brownish fat, in rodents. Thirty‐three human subjects (16 runners, 17 nonrunners) were measured for: resting energy expenditure (REE), body composition, VO_2_ Peak test, [irisin]_p_, and plasma metabolic profile. Nine female nonrunners then participated in a 10‐week supervised 5 km training program and tested after the race. Two runners underwent ^18^F‐FDG‐PET scans to quantify brown fat. No gender or age (28 ± 10 years) differences noted between matched cohorts. Runners averaged 58 ± 26 miles/week for 13 ± 6 years and had lower bodyweight (63 vs. 88 kg; *P* < 0.001), BMI (21 vs. 30 kg/m^2^; *P* < 0.0001), triglycerides (58 vs. 123 mg/dL; *P* < 0.01), total (white) fat (14 vs. 32%; *P* < 0.0001), and had higher VO_2_ Peak (63 vs. 34 mL/kg‐min; *P* < 0.0001) and HDL (65 vs. 48 mg/dL; *P* < 0.01) compared with nonrunners. [Irisin]_p_ was lower in runners versus nonrunners both before (179 vs. 197 ng/mL; NS) and after (207 vs. 226 ng/mL; NS) the VO_2_ Peak test. Significant (*P* < 0.05) positive correlations were noted between [irisin]_p_ versus BMI (*r*^2^ = 0.15), triglycerides (*r*^2^ = 0.40), and total body fat(g) (*r*^2^ = 0.24) with a significant negative correlation between [irisin]_p_ versus respiratory quotient (*r*^2^ = 0.33). Total lean mass significantly correlated with REE (*r*^2^ = 0.58) while total fat mass inversely correlated with VO_2_ Peak (*r*^2^ = 0.64). Nonrunners had lower [irisin]_p_ after completion of the training program (194 vs.181 ng/mL; pre‐ to post‐training; *P* > 0.05). Neither runner selected for ^18^F‐FDG‐PET scans had brown fat. Runners demonstrated significantly healthier metabolic and body composition profiles compared with nonrunners. None of these favorable exercise effects were positively associated with [irisin]_p._.

## Abstract (precis)

Well‐trained and elite runners had no detectable brown fat and lower plasma irisin levels compared to healthy, sedentary, age and gender‐matched overweight individuals. The metabolic health benefits of regular running were also not associated with elevated plasma irisin levels. Plasma irisin levels decreased after a 10‐week 5 km running program in overweight sedentary females.

## Introduction

Habitual running reduces disability compared with nonrunning controls, as demonstrated by a longitudinal study that tracked runners over two decades (Chakravarty et al. 2008). Furthermore, runners demonstrated 30% lower adjusted all‐cause mortality and 45% lower cardiovascular mortality rates compared with nonrunning controls, as documented in a prospective study involving 55,137 adults (Lee et al. 2014a). Unfortunately, half of all participants who commence regular exercise programs drop out within the first 6 months and never attain the health benefits associated with sustained aerobic fitness (Herring et al. 2014). Therefore, with the current (2011–2012) National Health and Nutrition Evaluation Survey data estimating the prevalence of obesity for US adults at 34.9% (68.5% are overweight and obese) (Ogden et al. 2014), recent discovery of the elusive “exercise hormone”, irisin, has generated avid scientific, medical and pharmaceutical interest.

Irisin is a circulating 112 amino‐acid extracellular dimer fragment of fibronectin type III domain containing protein 5 (FNDC5) that signals the conversion of white subcutaneous fat into metabolically active beige fat (Bostrom et al. 2012). Exercising muscle activates peroxisome proliferator‐activated receptor ɣ coactivator‐1*α* (PGC‐1*α*) that then triggers FNDC5 protein expression within muscle cell membranes (Bostrom et al. 2012). Subsequent proteolytic cleavage of the N‐terminal extracellular FNDC5 dimer fragment (irisin) into the circulation then triggers the “browning” of subcutaneous fat via upregulation of uncoupling protein 1 (UCP1). It is hypothesized that this “browning” of storage fat explains the favorable body composition and metabolic profiles characteristic of regular exercisers, such as endurance runners, via augmentation of resting energy expenditure (REE) and improvement of diet‐induced insulin resistance.

To date, the irisin messenger sequence between muscle and fat tissue appears valid in rodents, but equivocal in humans. Bostrom et al. (2012) documented a twofold increase in [irisin]_p_ in eight healthy middle‐aged men after 10 weeks of supervised aerobic training. However, this increase in basal [irisin]_p_ has yet to be supported by independent investigators who conversely report either a decrease (Hecksteden et al. 2013; Norheim et al. 2014) or no change (Kurdiova et al. 2014) in [irisin]_p_ following aerobic (Hecksteden et al. 2013; Kurdiova et al. 2014; Norheim et al. 2014) and strength (Hecksteden et al. 2013) training programs. These conflicting human data are thought to result from: lack of validation between commercially available assays used by independent laboratories (Sanchis‐Gomar et al. 2014); mutation of the FNDC5 start codon in humans compared with rodents (Raschke et al. 2013); ambiguity with regards to quantitation of extracellular FNDC5 protein after cleavage of irisin (Erickson 2013); and/or evidence suggesting that [irisin]_p_ is an adipokine (in addition to a myokine) (Roca‐Rivada et al. 2013). Cross‐sectional human studies also paradoxically demonstrate positive correlations between [irisin]_p_ and fat mass (Stengel et al. 2013; Crujeiras et al. 2014), body mass index (BMI) (Huh et al. 2012; Park et al. 2013; Stengel et al. 2013; Crujeiras et al. 2014), insulin resistance (Park et al. 2013), and triglycerides (Park et al. 2013). Thus, the favorable body composition and metabolic adjustments from exercise‐induced irisin secretion and augmentation of REE have yet to be convincingly validated in humans.

We wished to extend the current spectrum of understanding from unhealthy (Park et al. 2013; Stengel et al. 2013; Wen et al. 2013), middle‐aged (Bostrom et al. 2012; Hecksteden et al. 2013; Park et al. 2013; Crujeiras et al. 2014), populations toward a healthier extreme: endurance runners. Thus, the purpose of this investigation was to: (1) Assess plasma irisin concentrations ([irisin]_p_) in well‐trained runners and nonrunners before and after a VO_2_ Peak test; (2) Examine relationships between [irisin]_p_ versus REE, body composition, blood glucose, and lipid parameters; and (3) Assess changes in [irisin]_p_ in nonrunners before and after a 5 km run/walk training program. We hypothesized that highly trained runners would epitomize the classic irisin phenotype, which would be characterized by: elevated [irisin]_p_ high REE, low fat mass, favorable lipid panel, and robust depots of metabolically active beige fat (determined through positron emission tomography scans). We further hypothesized that nonrunners would demonstrate improvements in body composition, metabolic parameters and REE following 10 weeks of supervised aerobic training that were associated with favorable changes in [irisin]_p_.

## Materials and Methods

### Subjects

Thirty‐three healthy (no chronic medical condition requiring regular prescription medication) habitual runners (>50 km/week running for >3 months) and nonrunners (<60 min endurance activity for >3 months) between the ages of 18–50 years were recruited to participate in this trial. Sixteen runners (eight male, eight females) were age and gender‐matched with nonrunners (eight males, nine females). All female runners were tested during the follicular phase of their menstrual cycle. Informed written consent was obtained prior to participation. Phase I and II protocols were approved by Oakland University's Institutional Review Board (IRBNet #394700 and #471164, Phase I and II, respectively). Two runners (one male; one female) then underwent pilot cold‐stress^18^F‐fluorodeoxyglucose positron emission/computed tomography (^18^F‐FDG‐PET/CT) scans as part of a separate research study conducted at the Children's Hospital of Michigan PET Center and approved by Wayne State University's IRB (Brown Fat PET study).

### Experimental protocols

#### Phase I: runners versus nonrunners

All 33 participants presented to the laboratory after a minimum 4‐h fast. Weight and height were measured using a weight beam eye‐level physicians scale (Detecto, Webb City, MO). REE was measured via indirect calorimetry, using a metabolic canopy (VIASYS Vmax Encore, CareFusion, Loma Linda, CA). For the REE test, subjects were allowed 5–10 min to achieve steady‐state while resting comfortably in a recumbent position in a dimly lit room. REE and respiratory quotient (RQ) were averaged over a 15‐min assessment period. Next, in this recumbent position, 10 mL of venous blood was withdrawn from an antecubital vein for pre‐exercise measurement of plasma lipids, glucose, [irisin]_p_, and protein. After venipuncture, body composition was assessed using a dual energy X‐ray absorptiometry scan (Hologic Discover A, Boston, MA). A treadmill running test (VO_2_ Peak) was then conducted to determine aerobic fitness (TrueOne 2400, Parvo Medics, Sandy, UT). For the VO_2_ Peak test, runners started at an easy jog while nonrunners started at a comfortable walking speed. After 1 min, the treadmill speed increased 0.5 mph every minute until subjects could no longer keep pace with the treadmill (volitional exhaustion). After completion of the VO_2_ Peak treadmill test, all subjects returned to the recumbent position to allow re‐equilibration of body fluid compartments. After 15 min, another 10 mL of venous blood was withdrawn for postexercise assessment of plasma lipids, glucose, [irisin]_p_, and protein. Room temperature was maintained between 22–24°C.

#### Phase II: training trial

Within 5 days of baseline Phase I testing (above), nine female nonrunners participated in a 10‐week supervised run/walk 5 km training program. Phase II participants met three times per week and followed a freely available online training program designed to prepare beginners to finish a 5 km race comfortably (http://www.active.com/running/articles/how_to_run_your_first_5k?page=2). Within 5 days of completion of the target 5 km race, all Phase II runners repeated the Phase I testing protocol.

### Biochemical measures

Total cholesterol, high density lipoproteins (HDL), triglycerides, blood glucose, aspartate transaminase (AST), and alanine transaminase (ALT) were measured in whole blood (low density lipoproteins [LDL] and very low density lipoproteins [VLDL] were calculated) within 10 min of venipuncture, using a Lipid Panel Plus cartridge/Piccolo Xpress analyzer (Abaxis, Union City, CA). Irisin was measured in aprotinin‐treated plasma, using a commercially available enzyme immunoassay kit (EK‐067‐29, Phoenix Pharmaceuticals, Burlingame, CA). The Phoenix Pharmaceutical ELISA kit has been previously validated against western blotting (Wen et al. 2013) and mass spectrometry (Lee et al. 2014b), with a detection range between 0.1 and 1000 ng/mL. All plasma was stored at −80°C until analysis could be performed as a single batch, within 9 months of collection. Changes in plasma volume (PV) were estimated by comparing pre‐ and post‐VO_2_Peak measurements of plasma protein using a clinical refractometer (Schuco Clinical Refractometer 5711‐2020, Tokyo, Japan) according to a method described previously (Stricker 1968).

### Cold‐stress fludeoxyglucose (18F) positron emission tomography/computed tomography (^18^F‐FDG‐PET/CT) scans

Thermoregulatory challenge was applied using a specialized whole‐body garment through which subjects were exposed to a cold temperature stimulus. To achieve maximal stimulation, each subject was cooled until he or she was close to shivering and then the skin temperature was raised by 0.5°C and held steady thereafter. The tube suit cooling garment incorporated a network of small‐diameter plastic tubing (Allen Vangard, Inc., Ottawa, CA) through which temperature‐controlled cold water (2–4°C) was circulated. The effects of these exogenous temperature stressors on body temperature was monitored using a GaAs crystal sensor located at the tip of an optical fiber cable (OpSense, Inc., Quebec City, CA). This approach relies on the temperature dependence of the energy band gap of GaAs semiconductor crystal. The GaAs sensor is opaque for wavelengths below the bandgap and transparent for wavelengths above the energy band gap. The sensor was taped to the skin at the location of the left rib cage. This location was selected on the basis of proximity to important anatomical features (close to the pulmonary blood vessels which are possibly the most representative sites for body core temperature) and the ability to consistently place the sensors based on those anatomical landmarks.

Activated brown adipose tissue (BAT) in supraclavicular fat depots was considered present if there were areas of tissue that were more than 5 mm in diameter, had the CT density of adipose tissue (−250 to −50 Hounsfield units (HU)), and had a maximal standardized uptake value (SUV) of FDG of at least 2.0. This cutoff represented more than 2 SD above the maximal SUV seen in typical depots of white adipose tissue. BAT volume was determined by thresholding both the CT image volume (−250 < HU < −50) and the FDG volume (SUV > 2.0) and then applying the logical AND operation to the two masks, followed by removal of all areas that were smaller than 0.125 cm^3^. Using this definition of activated BAT, no voxel survived the thresholding operation. The PET protocol and quantification of brown adipose tissue has been described previously (Muzik et al. 2013).

### Statistical analyses

Unpaired *t*‐tests were chosen to express differences between group (runners vs. nonrunners) and gender (male vs. female) within each group, after 2‐way ANOVA tests revealed no significant interaction effects between group x gender for any measured variable (Phase I testing). Paired *t*‐tests were utilized to evaluate pretraining versus post‐training changes in those nonrunners who completed the 10‐week run/walk training program (Phase II). Linear regression analyses (Pearson's) were performed to assess relationships between variables. Statistically significant alpha level was set a priori at *P* < 0.05. All data presented as mean ± SD.

## Results

### Phase I: runners versus nonrunners

Eight male (109 ± 42 km/week; 11 ± 5 years running) and eight female (76 ± 38 km/week; 14 ± 9 years running) runners were age and gender‐matched with nonrunners. When males and females were combined, runners demonstrated significantly lower body weight and total body fat along with higher aerobic fitness (VO_2_ Peak) compared to nonrunners (Table 2). There were gender differences in both the runner and nonrunner groups with respect to total lean tissue, % total body fat, bone mineral content (BMC), and measured REE (Table 1).

**Table 1.** Demographic, body composition, aerobic fitness, and resting metabolism measurements obtained for both runners and nonrunners.

Variable (units)	Runners Mean ± SD			Nonrunners Mean ± SD		
	Male (min–max) *n*=8	Female (min–max) *n*=8	Combined (min–max) *N*=16	Male (min–max) *n*=8	Female (min–max) *n*=9	Combined (min–max) *N*=17
Age (years)	30.1 ± 11.0 (19 – 45)	30.4 ± 11.6 (20 – 48)	30.3 ± 10.9 (19–48)	29.8 ± 9.9 (20–48)	23.4 ± 6.9 (18–41)	26.4 ± 8.8 (18–48)
BMI (kg/m)	22.4 ± 2.4^aa^ (20–26)	20.3 ± 1.7^aaaa^ (17–23)	21.4 ± 2.3^aaaa^ (17–26)	29.8 ± 7.5 (20–43)	30.6 ± 4.9 (25–41)	30.2 ± 6.0 (20–43)
Weight (kg)	69.9 ± 8.4^aa,bb^ (61–85)	56.6 ± 8.3^aa^ (43–68)	63.2 ± 10.6^aa^ (43–85)	95.5 ± 23.5 (63–126)	81.6 ± 17.7 (67–125)	88.1 ± 21.2 (63–126)
Total Fat Tissue (kg)	6.9 ± 2.4^aaaa,bbb^ (4–11)	10.8 ± 3.6^aaa^ (6–15)	8.8 ± 6.6^aaaa^ (4–15)	26.1 ± 14.2 (12–47)	30.0 ± 9.9 (22–54)	28.1 ± 11.9 (12–54)
Total Lean Tissue (kg)	59.5 ± 6.5^bbbb^ (54–72)	43.3 ± 5.4 (35–50)	51.4 ± 10.2 (35–72)	65.2 ± 10.0^bbb^ (47–81)	47.8 ± 7.3 (41–65)	56.0 ± 12.3 (41–65)
Bone Mineral Content (kg)	2.54 ± 0.27^bbb^ (2.0–3.0)	1.97 ± 0.23 (1.8–2.4)	2.26 ± 0.39 (1.8–3.0)	2.61 ± 0.37^b^ (2.2–3.4)	2.14 ± 0.31 (1.7–2.6)	2.36 ± 0.41 (1.7–3.4)
% Body Fat	9.8 ± 2.7^aaaa,b^ (7–15)	18.9 ± 4.0^aaaa^ (12–24)	14.4 ± 5.9^aaaa^ (7–24)	26.1 ± 8.2^bb^ (18–39)	36.9 ± 3.8 (32–44)	31.8 ± 8.2 (18–44)
VO_2_ Peak (ml/kg‐min)	70.5 ± 9.6^aaaa, bbbb^ (57–83)	53.6 ± 5.1^aaaa^ (47–59)	62.6 ± 11.5^aaaa^ (47–83)	37.3 ± 8.9 (25–48)	30.8 ± 5.2 (24–39)	33.9 ± 7.7 (24–48)
Peak Treadmill Speed (mph)	13.2 ± 1.8^aaaa, bb^ (11–16)	10.4 ± 1.4^aaaa^ (9–12)	11.8 ± 2.0^aaaa^ (9–16)	7.6 ± 1.8 (5–10)	6.7 ± 0.9 (6–8)	7.1 ± 1.4 (5–10)
Maximum Heart Rate (bpm)	184.9 ± 10.1 (170–197)	188.6 ± 8.1 (176–198)	186.9 ± 9.0 (170–198)	191.1 ± 19.0 (149–211)	189.2 ± 0.3 (172–205)	190.1 ± 14.1 (149–211)
Measured REE (kcal/day)	1781.8 ± 107.1^bbb^ (1466–2027)	1436.4 ± 107.1 (1312–1556)	1609.1 ± 235.6 (1312–2027)	2059.9 ± 542.4^b^ (1405–3129)	1570.9 ± 241.6 (1438–2065)	1801.0 ± 470.3 (1405–3129)
RQ	0.80 ± 0.05 (0.7–0.9)	0.81 ± 0.07 (0.7–0.9)	0.80 ± 0.06 (0.7–0.9)	0.76 ± 0.04 (0.7–0.8)	0.78 ± 0.09 (0.7–1.0)	0.77 ± 0.07 (0.7–1.0)

^a^*P* < 0.05; ^aa^*P* < 0.01; ^aaa^*P* < 0.001; ^aaaa^*P* < 0.0001 between runners versus nonrunners.

^b^*P* < 0.05; ^bb^*P* < 0.01; ^bbb^*P* < 0.001; ^bbbb^*P* < 0.0001 between males versus females within cohort.

For the biochemical measurements, runners as a combined group (males and females) demonstrated significantly higher pre‐VO_2_Peak HDL, AST and post‐VO_2_ Peak blood glucose levels as well as lower pre‐VO_2_ Peak triglycerides and VLDL when compared to nonrunners (Table 2). There were no significant differences in pre‐VO_2_ Peak (resting) or Post‐VO_2_ Peak (stimulated) [irisin]_p_, with a trend for higher [irisin]_p_ in nonrunners compared to runners. Nonstatistically significant exercise‐induced increases in [irisin]_p_ were shown in both runners and nonrunners following the VO_2_ Peak test (Δ: postexercise minus pre‐exercise), even when corrected for plasma volume change (Table 2).

**Table 2.** Blood chemistry variables measured pre‐VO_2_ Peak test (resting), unless otherwise specified as post‐VO_2_ Peak test (exercise stimulated) or the change (Δ*: post‐VO*_*2*_ *Peak test minus pre‐VO*_*2*_ *Peak test value)*.

Variable (units)	Runners Mean ± SD			Nonrunners Mean ± SD		
	Male (min to max) *n*=8	Female (min to max) *n*=8	Combined (min to max) *N*=16	Male (min to max) *n*=8	Female (min to max) *n*=9	Combined (min to max) *N*=17
Cholesterol (mg/dL)	166.0 ± 24.8 (137 to 217)	176.8 ± 32.0 (144 to 227)	171.4 ± 28.2 (137 to 227)	165.0 ± 42.9 (105 to 226)	156.3 ± 26.1 (126 to 198)	160.4 ± 34.1 (105 to 226)
HDL (mg/dL)	59.4 ± 15.8 (38 to 76)	70.8 ± 12.5^aa^ (49 to 87)	65.1 ± 15.0^aa^ (38 to 87)	46.6 ± 15.7 (26 to 71)	49.1 ± 18.4 (30 to 82)	47.9 ± 16.7 (26 to 82)
LDL (mg/dL)	95.3 ± 22.4 (54 to 130)	94.3 ± 25.8 (62 to 137)	94.8 ± 23.4 (54 to 137)	92.7 ± 32.5 (52 to 136)	83.9 ± 14.5 (67 to 109)	88.0 ± 24.3 (52 to 136)
VLDL (mg/dL)	11.3 ± 3.9 (7 to 19)	12.0 ± 3.0^a^ (8 to 16)	11.6 ± 3.4^aa^ (7 to 19)	25.0 ± 24.3 (9 to 84)	23.2 ± 12.0 (8 to 38)	24.5 ± 18.2 (8 to 84)
Triglycerides (mg/dL)	56.0 ± 19.5 (36 to 95)	60.4 ± 14.5 (39 to 79)	58.2 ± 16.8^aa^ (36 to 95)	129.6 ± 121.9 (45 to 422)	116.7 ± 60.2 (38 to 192)	122.8 ± 29.7 (38 to 422)
ALT (U/L)	31.9 ± 7.8^b^ (25 to 44)	21.5 ± 9.6 (7 to 40)	26.7 ± 10.0 (7 to 44)	32.3 ± 20.6 (12 to 69)	23.6 ± 9.5 (12 to 35)	27.7 ± 15.8 (12 to 69)
AST (U/L)	41.5 ± 10.6 (25 to 59)	32.5 ± 5.8 (23 to 39)	37.0 ± 9.5^a^ (23 to 59)	32.3 ± 9.0 (24 to 48)	27.8 ± 7.9 (17 to 42)	29.9 ± 8.5 (17 to 48)
Pre‐VO_2_ peak glucose (mg/dL)	96.0 ± 4.0 (89 to 101)	96.4 ± 5.8^a^ (87 to 105)	96.2 ± 4.8 (87 to 105)	100.4 ± 8.5^bb^ (86 to 113)	89.7 ± 6.5 (89 to 99)	94.7 ± 9.1 (86 to 113)
Post‐VO_2_ peak glucose (mg/dL)	133.8 ± 11.1^aa^ (117 to 153)	125.8 ± 23.0^a^ (96 to 193)	129.8 ± 17.9^aaa^ (96 to 193)	106.9 ± 19.1 (87 to 140)	103.3 ± 12.6 (92 to 124)	105.1 ± 15.8 (87 to 140)
Pre‐VO_2_ peak irisin (ng/mL)	177.8 ± 33.1 (128 to 228)	180.0 ± 41.0 (103 to 223)	178.9 ± 36.0 (103 to 228)	200.0 ± 69.0 (116 to 345)	193.9 ± 35.8 (109 to 234)	196.8 ± 52.3 (109 to 345)
Post‐VO_2_ peak irisin (ng/mL)	191.8 ± 13.9 (174 to 217)	221.9 ± 30.9 (161 to 270)	206.9 ± 27.9 (161 to 270)	224.2 ± 72.9 (162 to 378)	228.2 ± 21.6 (179 to 248)	226.2 ± 52.0 (162 to 378)
Δ Irisin (ng/mL)	14.0 ± 23.5 (−13 to 53)	42.0 ± 59.9 (−14 to 167)	28.0 ± 46.3 (−14 to 167)	24.3 ± 42.9 (−46 to 77)	39.3 ± 19.0 (22 to 70)	31.8 ± 32.9 (−46 to 77)
Δ Plasma volume (%)	6 ± 6 (0 to 19)	3 ± 4 (−6 to 6)	4 ± 5 (−6 to 19)	5 ± 4 (0 to 12)	5 ± 4 (0 to 11)	5 ± 4 (0 to 12)
Δ Irisin PV corrected (ng/mL)	2.4 ± 17.2 (−19 to 27)	36.0 ± 57.0 (−21 to 154)	19.2 ± 44.2 (−21 to 154)	12.6 ± 37.8 (−54 to 48)	26.9 ± 20.4 (2 to 70)	14.0 ± 23.5 (−54 to 70)

^a^*P* < 0.05; ^aa^*P* < 0.01; ^aaa^*P* < 0.001; ^aaaa^*P* < 0.0001 between runners versus nonrunners.

^b^*P* < 0.05; ^bb^*P* < 0.01; ^bbb^*P* < 0.001; ^bbbb^*P* < 0.0001 between males versus females within cohort.

**Figure 1.**
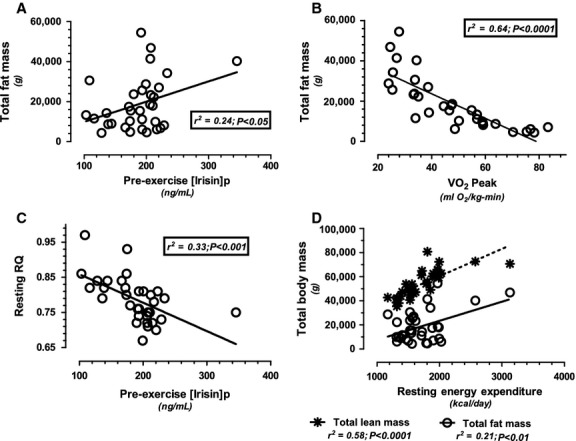
Statistically significant correlations (*r*^2^) when data from both runners and nonrunners were combined (*N* = 33) between: total fat mass versus pre‐exercise plasma irisin concentration (1A); total fat mass versus VO_2_ Peak (1B); resting RQ versus pre‐exercise plasma irisin concentration (1C); and total lean and fat mass versus resting energy expenditure (1D).

When all data were combined (*N* = 33), resting [irisin]_p_ was positively correlated with BMI, total cholesterol, VLDL, triglycerides, trunk fat (Table 3), and total fat mass (Fig. 1A), while negatively correlated with RQ (Fig. 1C). Total lean mass was positively correlated with measured REE (Fig. 1D). Total fat mass was positively correlated with REE (Fig. 1D), BMI, VLDL, and triglycerides while negatively correlated with HDL (Table 3) and VO_2_ Peak (Fig. 1B).

**Table 3.** Statistically significant linear correlations (*r*^2^) between pre‐exercise plasma irisin concentration, total lean body mass, and total body fat mass (*N* = 33) versus demographic, body composition or biochemical measurements that were obtained pre‐VO_2_ Peak test.

Variable	BMI (kg/m^2^)	Cholesterol (mg/dL)	HDL (mg/dL)	VLDL (mg/dL)	Triglycerides (mg/dL)	ALT (U/L)	Trunk fat (kg)
Pre‐irisin (ng/mL)	0.15*	0.26**	0.00	0.40****	0.40****	0.07	0.16*
Total lean tissue (kg)	0.26**	0.00	(−)0.05	0.06	0.06	0.28***	0.15*
Total fat tissue (kg)	0.88****	(−)0.01	(−)0.20**	0.28**	0.28**	0.12	0.98****

*P < 0.05; **P < 0.01; ***P < 0.001; ****P < 0.0001.

(−) designates that the correlation is negatively directed (inverse).

### Phase II: training trial

**Figure 2.**
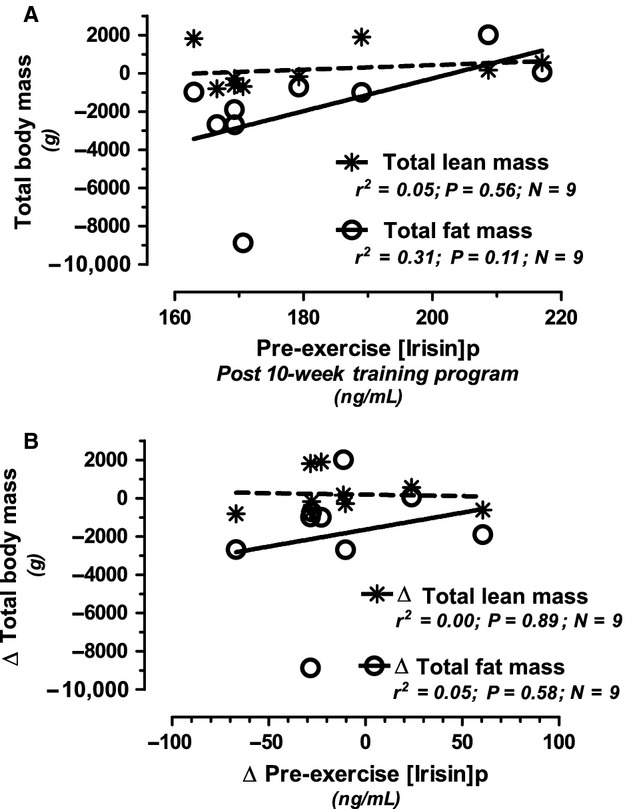
Nonstatistically significant relationships in the nine female nonrunners (Phase II) between the change (Δ: post‐training minus pretraining) in total lean and fat mass versus pre‐exercise plasma irisin concentrations when measured both: after the 10‐week training program (post‐training; 2A); or expressed as the change (Δ: post‐training minus pretraining) in pre‐VO_2_ Peak (resting) plasma irisin concentration (2B).

Nine female nonrunners successfully completed the supervised 10‐week run/walk 5 km training program. Two male nonrunners started the training program but dropped out within the first 4 weeks (82% completion rate overall). Although the average trend favored a net ~2 kg weight loss over the training period, six females lost weight (Losers) while three females either gained or maintained weight (Gainers). [Irisin]_p_ tended to decrease following the training program, when compared to pretraining levels, despite (nonsignificant) gains in aerobic capacity. This pre‐ to post‐training decrease in [irisin]_p_, was noted both before and after the VO_2_ Peak test, regardless of weight loss or gain. There were no statistically significant correlations noted between resting [irisin]_p_ versus the change in either lean mass or fat mass immediately post‐training (Fig. 2A) or when expressed as a change (Fig. 2B). There was a significant difference in post‐training pre‐VO_2_ Peak [irisin]_p_ between Losers versus Gainers (169.7 vs. 204.9 ng/mL; *P* < 0.001, respectively) with the Gainers demonstrating higher [irisin]_p_ levels before and after the training program. The only other statistically significant difference between pretraining versus post‐training variables or between Losers versus Gainers was seen in ALT, which decreased more in Losers compared to Gainers after the training program (Table 4).

**Table 4.** Changes (Δ: post‐training minus pretraining) in body composition, aerobic fitness, resting energy expenditure, and blood chemistry variables for the nine female nonrunners who completed the 5 km training program (Phase II). “Losers” refer to those females who lost weight after the 10‐week walk/run program (*n* = 6). “Gainers” refer to those females who maintained or gained weight after the 10‐week walk/run program (*n* = 3).

Variable (units)	Total (min to max) *N*=9	Losers *n*=6	Gainers *n*=3
Δ Weight (kg)	−1.8 ± 3.9 (−11 to 2)	−3.5 ± 3.9	1.4 ± 1.2
Δ Total fat tissue (kg)	−1.9 ± 3.0 (−9 to 2)	−3.0 ± 3.0	0.3 ± 1.5
Δ Total lean tissue (kg)	0.2 ± 1.0 (−0.8 to 1.9)	−0.1 ± 1.0	0.9 ± 0.9
Δ Bone mineral content (g)	−16.6 ± 26.2 (−55 to 39)	−12.2 ± 30.8	−25.7 ± 13.8
Δ VO_2_ peak (ml/kg‐min)	2.2 ± 3.0 (−1 to 8)	2.6 ± 3.2	1.3 ± 2.8
Δ Measured REE (kcal/day)	8.9 ± 175.6 (−182 to 254)	−14.8 ± 169.9	56.3 ± 214.8
Δ Cholesterol (mg/dL)	6.9 ± 15.3 (−14 to 31)	17.7 ± 12.2	1.5 ± 14.6
Δ HDL (mg/dL)	3.3 ± 8.2 (−12 to 16)	6.3 ± 9.5	1.8 ± 8.0
Δ LDL (mg/dL)	6.4 ± 11.2 (−16 to 20)	9.7 ± 4.6	4.8 ± 13.5
Δ VLDL (mg/dL)	−2.8 ± 6.6 (115 to 3)	−4.7 ± 7.5	1.0 ± 2.0
Δ Triglycerides (mg/dL)	−14.6 ± 33.6 (−77 to 15)	−24.8 ± 37.5	6.0 ± 7.6
Δ ALT (U/L)	−4.0 ± 6.8 (−18 to 4)	−7.2 ± 6.0*	2.3 ± 2.1
Δ AST (U/L)	−3.3 ± 8.7 (−18 to 8)	−4.2 ± 10.8	−1.7 ± 2.1
Δ Glucose (mg/dL)	3.0 ± 4.4 (−4 to 12)	3.0 ± 8.2	3.0 ± 1.9
Δ Pre‐VO_2_ peak irisin (ng/mL)	−12.5 ± 36.2 (−29 to 61)	−17.0 ± 42.2	−3.6 ± 24.3
Δ Post‐VO_2_ peak irisin (ng/mL)	−44.4 ± 46.9 (−120 to 9)	−47.3 ± 55.7	−39.7 ± 37.9

* *P* < 0.05 between responders and nonresponders.

### ^18^F‐FDG‐PET/CT scans

**Figure 3.**
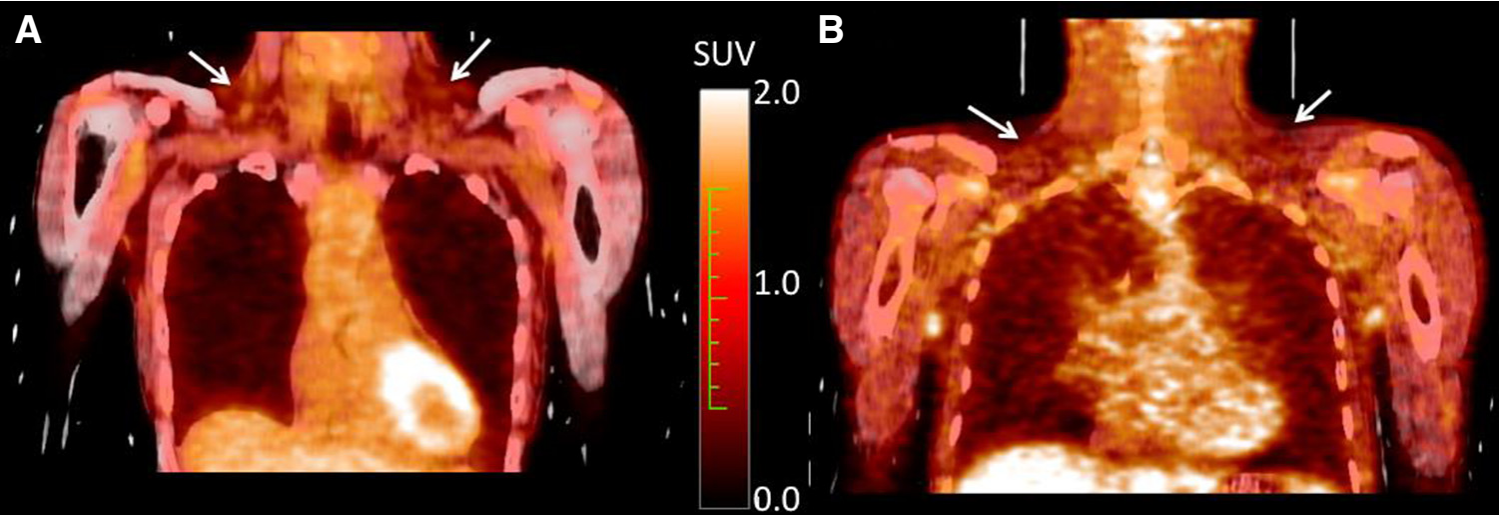
FDG PET/CT images of a female (A) and a male (B) runner show low FDG uptake in supraclavicular adipose tissue depots (white arrows). The maximal FDG SUV in these depots was determined as 1.5 in the female and 0.7 in the male runner.

One female and one male runner underwent cold‐stimulated ^18^F‐FDG‐PET/CT scans, to assess brown adipose tissue activation as part of a separate project. Possible activation of brown adipose tissue was assessed based on an FDG standard uptake value (SUV) threshold (SUV > 2.0) and presence of adipose tissue as determined by CT (HU < −50). No detectable activated brown fat was noted in either runner 1 (Fig. 3A: 48 year old female, 30 years running, 50 km/week running, SUV_max_ = 1.5, VO_2_ Peak 49 mL/kg‐min, BMI 17 kg/m^2^, body fat 14%, [irisin]_p_ 217 ng/mL pre and 229 ng/mL postexercise) or runner 2 (Fig. 3B: 24 year old male, 10 years running, 174 km/week running, SUV_max_= 0.7, VO_2_ Peak 84 mL/kg‐min, BMI 22 kg/m^2^, body fat 10%, [irisin]_p_ 166 ng/mL pre‐exercise and 192 ng/mL postexercise). The reported SUVs correspond to the location of supraclavicular fat depots defined based on HU values of (−250 < HU < −50).

## Discussion

These data do not support any positive association between [irisin]_p_ with favorable body composition profiles, healthy blood lipid parameters, or cold‐stimulated brown fat activation in fit and unfit humans. Furthermore, there was a tendency for [irisin]_p_ to decrease following a 10‐week supervised run/walk training program, despite increases in aerobic fitness in our cohort of healthy, young, and overweight females. This lack of a positive [irisin]_p_ response has been similarly reported in other prospective cohort training studies conducted in healthy humans (Hecksteden et al. 2013; Kurdiova et al. 2014; Norheim et al. 2014). To date, only the original Bostrom et al. (2012) study has demonstrated a significant (two‐fold) increase in circulating irisin concentrations following an aerobic training program in humans.

Investigations targeting FNDC5 gene expression within human muscle following both a 10‐week endurance training and an 11‐week strength training program also failed to demonstrate increases in FNDC5 gene expression (Raschke et al. 2013). Cross‐sectional studies revealed significant increases in muscle FNDC5 gene expression only in older (~55 years) active versus sedentary subjects (Timmons et al. 2012) and in heart failure patients (~67 years) with higher aerobic capacity, when subdivided into more fit (VO_2_ Peak ~17 mL/kg‐min) versus less fit (VO_2_ Peak ~11 mL/kg‐min) groups for statistical comparison (Lecker et al. 2012). Because circulating irisin is the cleaved, extracellular, polypeptide fragment of FNDC5, these upstream results strengthen a growing body of evidence suggesting that [irisin]_p_ is unrelated to the favorable metabolic or body composition effects associated with regular endurance exercise in healthy humans.

The runners in this study were well‐trained and highly fit, as demonstrated by mean peak oxygen uptakes (VO_2_ Peak) above the 90th percentile for both males (90th percentile: 55 mL/kg‐min; our cohort: 71 mL/kg‐min) (ACSM’s Guidelines for Exercise Testing and Prescription 2014a) and females (90th percentile 46 mL/kg‐min; our cohort: 54 mL/kg‐min) (ACSM’s Guidelines for Exercise Testing and Prescription 2014b). Of note, our male cohort included elite, professional, runners, with mean peak aerobic capacities representative of international competitors, such as the Swiss National Team (73 mL/kg‐min) (Marti and Howald 1985). Conversely, the nonrunners in this study had peak oxygen uptakes below the 20th percentile for both males (20th percentile: 38 mL/kg‐min; our cohort 37 mL/kg‐min) (ACSM’s Guidelines for Exercise Testing and Prescription 2014a) and females (20th percentile: 31 mL/kg‐min; our cohort: 31 mL/kg‐min) (ACSM’s Guidelines for Exercise Testing and Prescription 2014b). However, despite the wide disparity in aerobic fitness levels between runners and nonrunners, all of our subjects were healthy by design, with fasting blood lipid and glucose levels within the normal range. The only exception was pretraining HDL levels in the female nonrunners (49 mg/dL), with an overall mean value just below the biochemical range of “poor” (<50 mg/dL) (Table 2).

Even amongst “healthy” individuals, the runners demonstrated superior metabolic health with significantly higher HDL, lower triglycerides, and VLDL compared to the nonrunners. The male and female runners were also exceptionally lean, with significantly lower total body fat mass when compared to nonrunners. With regards to our main outcome measure, [irisin]_p_, none of the favorable aerobic fitness, metabolic health, or body composition profile measurements were positively correlated with either resting (pre‐VO_2_ Peak) or exercise stimulated (post‐VO_2_ Peak) [irisin]_p_. In fact, our mean [irisin]_p_ values tended to be lower in runners compared to nonrunners, both before and after the VO_2_ Peak test. Also in direct contrast with Bostrom et al. (2012) results, [irisin]_p_ decreased in our cohort of female nonrunners immediately following 10‐weeks of endurance training. Neither post‐training [irisin]_p_ or the pre‐ to post‐training change in [irisin]_p_ were linearly related to changes in total lean mass. This decrease in resting and stimulated [irisin]_p_ occurred regardless of body weight loss or gain and despite nonsignificant increases in aerobic fitness and improvements in metabolic health (increased HDL and decreased triglycerides). Thus, our collective findings from Phase I and II trials were opposite of what was expected, assuming that irisin receptor number, sensitivity, and clearance rate were equivalent in runners and nonrunners before and after the training program. Additionally, resting [irisin]_p_ was positively correlated with BMI, total cholesterol, triglycerides, VLDL, trunk, and total fat mass while negatively correlated with resting RQ. Similar relationships between [irisin]_p_ versus increasing adiposity and poorer metabolic health have been verified previously (Huh et al. 2012; Park et al. 2013; Stengel et al. 2013; Crujeiras et al. 2014) and thereby strengthen growing support that [irisin]_p_ neither mimics nor augments the beneficial effects of regular endurance exercise in humans.

Resting energy expenditure was not related to [irisin]_p_ in our cohort of runners and nonrunners, as originally expected. Total lean mass explained 58% of the variance in REE. Nonrunners who gained weight after the 10‐week training program (Gainers) demonstrated gains in lean tissue, which were accompanied by increases in REE. Although total lean mass was not linearly related to [irisin]_p_, on average, nonrunners tended to have greater lean mass, REE, and [irisin]_p_ compared to runners, but these differences were not statistically significant. These trends in magnitude support irisin as a myokine, stimulated in both runners and nonrunners by high intensity exercise even after correcting for plasma volume change. Both submaximal exercise and shivering have also been shown to induce transient increases in [irisin]_p_ further supporting it's myokine lineage (Huh et al. 2012; Kraemer et al. 2014; Lee et al. 2014b). However, although it appears that irisin is released by muscular contraction, plasma irisin levels do not seem to be linearly related with increases in REE, total lean mass or any beneficial metabolic or body composition effects associated with aerobic fitness.

Unlike total lean mass, significant positive relationships were noted between resting [irisin]_p_ versus total fat mass and resting RQ. Total fat mass explained 21% of the variance in REE and positively correlated with BMI, triglycerides and VLDL, while negatively correlated with HDL and VO_2_ Peak. For the nonrunners who completed the 10‐week training program, there was a nonstatistically significant trend for post‐training [irisin]_p_ to be positively correlated with changes in total fat mass and not lean mass as originally hypothesized. Thus, our data from both Phase I and Phase II trials suggest that [irisin]_p_ better reflects a white, not brown, adipose tissue lineage.

Lastly, because the metabolic and body composition benefits of irisin are related to the “browning” of subcutaneous white adipose tissue, we performed cold‐stress ^18^F‐FDG‐PET/CT scans (Muzik et al. 2013) in a pair of runners (one female and one male) deemed most likely to exhibit robust depots of beige or brown fat. The female was lean (BMI = 17 kg/m), had been running for >30 years and had higher than average [irisin]_p_ levels (217–229 ng/mL). The male was a professional runner, averaging >160 km/week running, and had the highest VO_2_ peak of the cohort (84 mL/kg‐min). Unexpectedly, neither runner had any detectable beige or brown fat activity within the regions of interest. Because cold‐induced postganglionic sympathetic nervous system (SNS) release of norepinephrine activates UCP1 in brown fat, we hypothesized that athletes would have an augmented capacity to secrete adrenaline (“sports adrenal medulla”) (Zouhal et al. 2008). We then hypothesized that chronic, repetitive, SNS stimulation from daily running would enhance the browning of subcutaneous fat and lead to greater cold‐stimulated brown fat activation in more seasoned runners, regardless of circulating plasma irisin levels. Circulating norepinephrine concentrations have also been shown to increase >20 times basal levels after a 400 m race and rise exponentially with increases in exercise intensity (Zouhal et al. 2008). Although we did not measure catecholamine's in this study, the maximum heart rates of 185–191 bpm seen in runners and nonrunners after the VO_2_ Peak test indirectly imply strong SNS activation, well above previous administration of ephedrine in another study which failed to induce brown fat activation, with pharmacologically driven heart rate increases of only ~10 bpm (Cypess et al. 2012). Previous studies using cold‐stress ^18^F‐FDG‐PET/CT also document high brown fat mass (>50 g) and activation in 96% of healthy males (van Marken Lichtenbelt et al. 2009) and 53% of females (Muzik et al. 2013). This high prevalence of brown fat in healthy humans makes our negative PET findings all the more curious, given the potential metabolic benefits of both running and brown fat (Ouellet et al. 2012; Chondronikola et al. 2014) which did not coexist in our two carefully chosen lean subjects.

### Limitations

**Figure 4.**
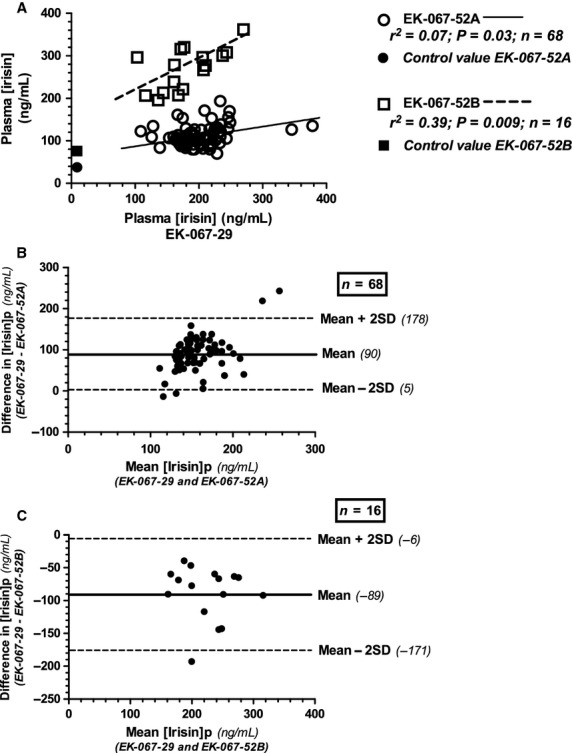
Statistical evaluation of the three Phoenix Pharmaceutical ELISA kits utilized in this study. Two kits (EK‐067‐52) had separate lot numbers (designated A and B) and analyzed as two separate batches. The values used in these final analyses were analyzed as a single batch using the latest available kit (EK‐067‐29). Linear regression analyses between the previous (EK‐067‐52) kits versus the current (EK‐067‐029) kit are shown (4A) along with Bland‐Altman plots (4B and 4C) illustrating mean differences.

The wide variability in circulating irisin levels reported in this study ‐ and in other published reports (Sanchis‐Gomar et al. 2014)‐ represents the biggest limitation in the interpretation of [irisin]_p_. Although the Phoenix Pharmaceutical ELISA kit has been previously validated against immunoblotting and mass spectrometry, there have been three generations of commercial assay kits (EK‐067‐16, EK‐067‐52, and EK‐067‐29). Our first samples were measured using two separate EK‐067‐52 kits, which differed in lot number. When a twofold difference was noted between in the mean values from the separate kits, all samples were subsequently reanalyzed using the newest generation assay (EK‐067‐29). Linear regression (Fig. 4A) and Bland‐Altman analyses (Fig. 4B and C) highlight the wide variability between the mean values (90 to −89) for the different ELISA kits and underscores the inconsistencies in measurement and reporting of [irisin]_p_ (Erickson 2013; Sanchis‐Gomar et al. 2014). The coefficient of variation (CV) for each kit was fairly similar and seemed to increase with increasing subject numbers: (EK‐067‐29; *N* = 84; CV = 0.24. EK‐067‐52A; *n* = 68; CV = 0.22; EK‐067‐52B; *n* = 16; CV = 0.18).

Another potential limitation was our 4‐h fasting period, which may not have been adequate to obtain truly fasted lipid and REE values. We choose the 4‐h fasting interval from a previous investigation (Haugen et al. 2003) showing that RMR differed by ~100 kcal/day when compared to an overnight fast. We also analyzed the REE data using the kcal/day “correction” factor and found that these data were not significantly altered between the subjects who participated in an overnight versus 4‐h fast (*n* = 7; 21%; data not shown).

### Perspectives and significance

The lack of a positive relationship between exercise, aerobic fitness, circulating irisin and the browning of fat highlights the potential difficulty translating evidence obtained in murine models directly to humans. Exercise training of healthy male Yucatan miniature swine pigs similarly did not elicit significant changes in muscle FNDC5 or circulating irisin levels after 16–20 weeks of regular treadmill running (Fain et al. 2013). Genomic sequencing reveals that humans contain an alternative start codon for FNDC5 (ATA) while rodents bear the highly conserved ATG start codon (Raschke et al. 2013). As such, annotated noncanonical ATA start sites demonstrate low‐translation efficiency. Only three human genes with an ATA start codon have been previously identified to translate full length proteins. In vitro data verify that only 1% of the full length FNDC5 protein is translated with an ATA start, which may explain the discrepancies between human versus rodent [irisin]_p_ data (Raschke et al. 2013). Unfortunately, swine were not included in these multispecies FNDC5 sequence alignments to corroborate these negative human and pig exercise training data.

In addition to the potential inconsistencies when translating animal data to human models, the vast majority of human exercise interventions target disease (inactive) and/or normal (active) populations. Investigations targeting exceptionally healthy populations, such as endurance athletes, are often overlooked when evaluating the favorable physiological adaptations associated with regular physical activity. While we typically construct our physiological understanding of dysregulation starting with disease (chronic inactivity), a simultaneous deconstruct of highly fit individuals (chronic physical activity) would anchor both sides of the spectrum. The athletes profiled in this investigation have maximized their physiological capacity to effectively respond to daily bouts of intense homeostatic challenge. As such, we feel that our Phase I and Phase II exercise testing sequence provides a useful “reality check” in the evolution of molecular pathways and targets which serve to explain and ‐ ultimately mimic – the health benefits of exercise as derived from in vitro and animal models.

## Acknowledgments

The authors thank Qin Xu from Georgetown University Medical Center's Endocrinology and Metabolism Laboratory for performing the irisin assays; Phoenix Pharmaceuticals, Inc., for donating the updated (EK‐067‐29) ELISA kits; Matt VanSumeren and Yousef Sirajeldon for the exercise laboratory assistance; and to our 33 enthusiastic subjects who were inspirational in our quest to verify whether exercise can be packaged into a pill.

## Conflict of Interest

None declared.
